# Identification of a Conserved Linear Epitope in SARS-CoV-2 Nucleocapsid Protein Recognized by Monoclonal Antibody N179

**DOI:** 10.3390/v18090949

**Published:** 2026-08-30

**Authors:** Zhoujun Su, Junhao Guo, Ning Li, Hefeng Zhou, Jun Ni, Yongjun Cao, Liping Peng, Min Shao, Haitao Li

**Affiliations:** 1 School of Bioengineering, Zhuhai Campus, Zunyi Medical University, Zhuhai 519041, China; 2 Department of Stomatology, Zhuhai Campus, Zunyi Medical University, Zhuhai 519041, China; 3 School of Pharmacy, Macau University of Science and Technology, Macau 999078, China

**Keywords:** SARS-CoV-2, nucleocapsid protein, monoclonal antibody, linear B-cell epitope, colloidal gold-based immunochromatographic assay, epitope mapping

## Abstract

We previously generated mouse monoclonal antibody N179 against the SARS-CoV-2 nucleocapsid (N) protein and developed a colloidal gold-based immunochromatographic test strip with a 2 ng/mL detection limit and 98% concordance with RT-qPCR. However, the precise epitope recognized by N179 remained undefined. Using GST-fused N protein truncation fragments, Western blotting, and ELISA, we mapped the linear antibody-reactive region to the C-terminal tail of the N protein and identified ^390^QTVTLL^395^ as the smallest reactive region under the truncation-mapping conditions used. Multiple sequence alignment of 11 representative SARS-CoV-2 N protein sequences showed complete conservation of this motif across the sequences analyzed. EMBOSS WATER analysis further demonstrated a perfect 6/6 match only in SARS-CoV-2; no identical sequence was found in six other human coronaviruses, four influenza viruses, or five bat coronaviruses. Computational structural analyses predicted that this region may be surface accessible, and all six residues exceeded the default BepiPred 3.0 threshold. These findings identify ^390^QTVTLL^395^ as the smallest reactive region defined by truncation mapping and show that this sequence is conserved among the representative SARS-CoV-2 lineages analyzed. Together with the sequence comparison and structural predictions, these results provide sequence-level information that may help explain the previously observed recognition profile of N179. This motif may serve as a reference for the selection and evaluation of diagnostic antibodies targeting conserved regions of the SARS-CoV-2 N protein.

## 1. Introduction

Rapid and accurate diagnostic testing remains important for timely case identification, clinical management, and SARS-CoV-2 surveillance [1]. Reverse transcription-quantitative PCR (RT-qPCR) is still regarded as the reference standard. However, its dependence on specialized laboratory facilities and trained personnel limits its application in point-of-care testing and large-scale screening settings [1,2]. Antigen rapid diagnostic tests (Ag-RDTs) based on lateral flow immunochromatography therefore provide a practical alternative owing to their operational simplicity, rapid turnaround time, and low cost [3,4,5].

The nucleocapsid (N) protein is one of the four major structural proteins of SARS-CoV-2 [6]. Because the N protein is abundant in viral particles and infected cells, highly immunogenic, detectable in respiratory specimens, and relatively conserved across viral lineages, it serves as a major target for SARS-CoV-2 antigen detection [7,8,9]. Nevertheless, the continued emergence of SARS-CoV-2 variants threatens the reliability of antibody-based diagnostic assays [10,11,12,13]. Mutations in the N protein, particularly those located within antibody-recognition epitopes, can diminish or even abolish antibody binding, thereby resulting in false-negative outcomes [11]. For instance, the D399N substitution in the B.1.429 variant substantially compromised the performance of the Quidel Sofia SARS Antigen FIA test, demonstrating that a single amino acid substitution can impair diagnostic sensitivity [14]. Similarly, antibodies targeting the N-terminal RNA-binding domain or the central dimerization domain can be disrupted by mutations within their respective epitopes [15]. These observations underscore the critical need to identify and characterize monoclonal antibodies that recognize highly conserved linear epitopes in the N protein [14,15].

We previously generated a mouse monoclonal antibody, N179, against the SARS-CoV-2 N protein and used it to develop a colloidal gold-based immunochromatographic test strip with a detection limit of 2 ng/mL and 98% concordance with RT-qPCR results [16]. Despite the diagnostic performance of this strip, the precise epitope recognized by mAb N179 remained undefined. Several questions thus remained unresolved: whether the antibody-reactive region is conserved among representative SARS-CoV-2 lineages, how its sequence compares with corresponding regions in selected related respiratory viruses, and what predicted molecular features may be associated with mAb N179 recognition. In this study, we mapped the linear epitope recognized by mAb N179 using GST-fused N protein truncation fragments, Western blotting, and ELISA validation. We further assessed the conservation of this epitope across representative SARS-CoV-2 variants, examined its sequence specificity among seven human coronaviruses, four influenza viruses, and five bat coronaviruses, and characterized its predicted structural features. Together, these analyses clarify the epitope-level basis of mAb N179 recognition, evaluate the conservation and sequence distinctiveness of the identified motif, and provide a possible sequence-level explanation for the previously observed specificity profile of the mAb N179-based assay.

## 2. Materials and Methods

### 2.1. Expression and Purification of Full-Length N Protein and Antibody Validation

The recombinant plasmid pET-28a(+)-CorN, previously constructed in our laboratory, was transformed into *Escherichia coli* BL21 (DE3) competent cells (Biomed, Beijing, China). Protein expression was induced with 0.5 mM IPTG at 16 °C for 16 h. The cells were harvested, resuspended in lysis buffer containing 50 mM Tris-HCl, 500 mM NaCl, 10 mM imidazole, and 1 mM PMSF at pH 7.4, and disrupted by ultrasonication. After centrifugation, the supernatant was collected for purification.

His-tagged recombinant N protein (CorN) was purified using an ÄKTA system equipped with a HisTrap Excel column (Cytiva, Uppsala, Sweden). After equilibration and washing with binding buffer, the target protein was eluted using a linear imidazole gradient from 10 to 500 mM. Peak fractions were collected, concentrated, and buffer-exchanged into PBS using a 10 kDa cutoff ultrafiltration device (Millipore, Burlington, MA, USA).

The purified protein was quantified by BCA assay (Beyotime, Shanghai, China) and analyzed by SDS-PAGE and Western blotting to verify its reactivity with mAb N179 (mouse IgG2a/κ), previously developed and stored in our laboratory [16]. Briefly, N179-producing hybridoma cells were established by fusing splenocytes from BALB/c mice immunized with recombinant SARS-CoV-2 N protein (CorN) with SP2/0-Ag14 myeloma cells, followed by indirect ELISA screening and limiting-dilution subcloning. For antibody production, the N179 hybridoma cells were intraperitoneally injected into pristane-pretreated BALB/c mice to generate ascitic fluid. In the present study, N179-containing ascitic fluid was used directly without further antibody purification and was stored at −20 °C until use. Protein samples were separated on a 12% SDS-PAGE gel and transferred onto a PVDF membrane (Millipore, Burlington, MA, USA). After blocking, the membrane was incubated with mAb N179 (1:10,000), followed by HRP-conjugated goat anti-mouse IgG (1:2000, Beyotime, Shanghai, China). Chemiluminescent signals were detected using an ECL kit (Beyotime, Shanghai, China).

### 2.2. Construction and Expression of Truncated GST Fusion Proteins

**Figure 1:**
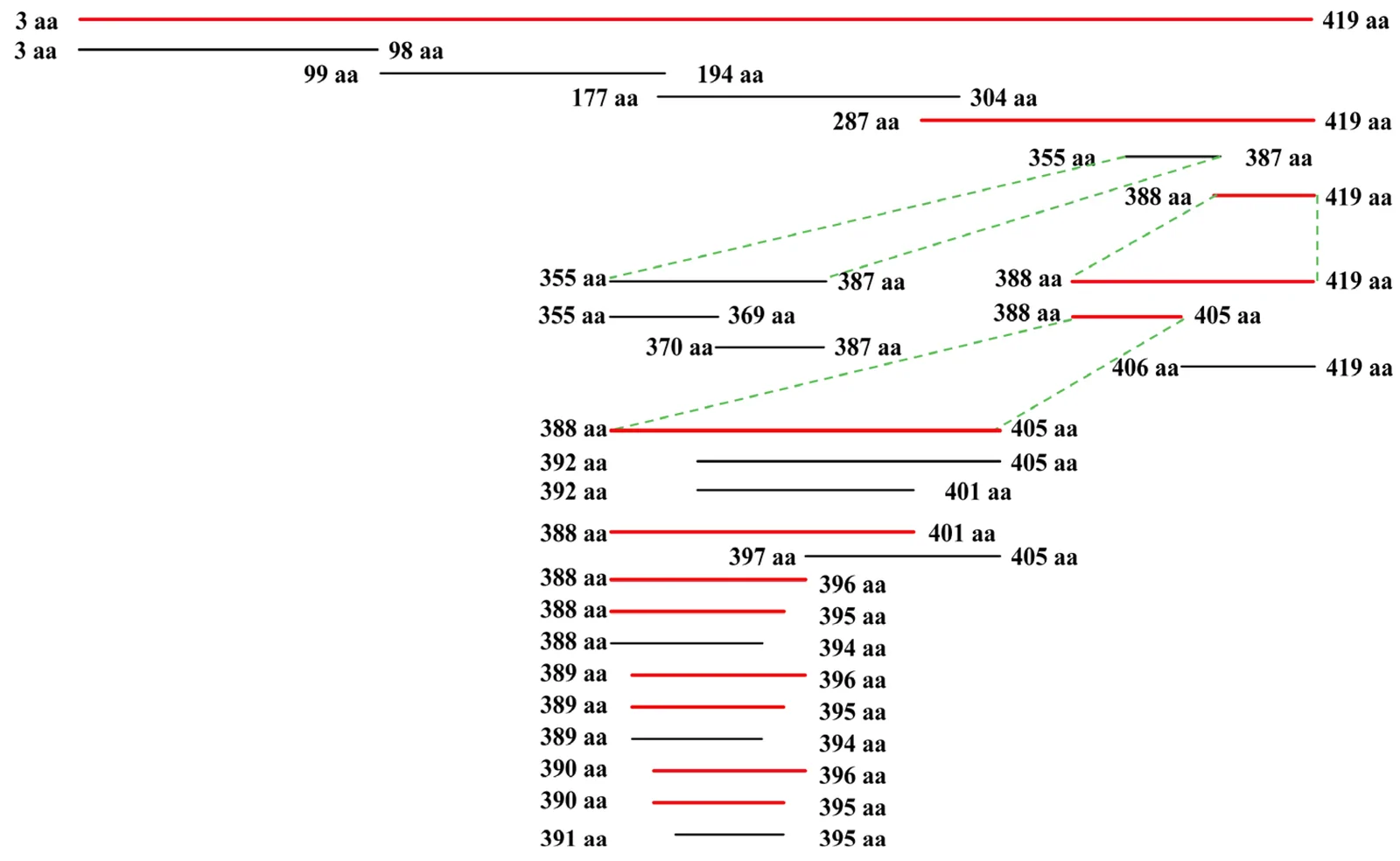
Schematic representation of the truncated SARS-CoV-2 N protein fragments used for epitope mapping. Amino acid positions are indicated for each fragment. The near-full-length N protein fragment (3–419 aa) and progressively truncated fragments were analyzed by Western blotting and ELISA. Red bars indicate fragments that showed positive reactivity with mAb N179. Green dashed lines are visual guide lines used to highlight the relationship between selected parent fragments and the corresponding shorter fragments (black bars) generated for further epitope mapping; they do not represent protein domains or structural features.

To localize the region recognized by mAb N179, primers were designed based on the SARS-CoV-2 reference genome sequence in GenBank (accession no. NC_045512.2; Table S1). Recombinant plasmids expressing a GST-fused near-full-length N protein fragment (3–419 aa) or truncated fragments covering different C-terminal regions were constructed (Figure 1). The pGEX-4T-3 vector was digested with *Bam*HI and *Not*I (New England Biolabs, Ipswich, MA, USA), and the amplified fragments were inserted by ligation or homologous recombination (Vazyme, Nanjing, China). All constructs were verified by Sanger sequencing.

Each recombinant plasmid was transformed into *Escherichia coli* BL21 (DE3) cells. Protein expression was induced with 0.5 mM IPTG at 16 °C for 16 h. Cells were harvested, resuspended in PBS containing 1 mM PMSF, and disrupted by ultrasonication. After centrifugation, GST-tagged proteins in the supernatants were purified using an ÄKTA system equipped with a GST affinity column (Smart-Lifesciences, Changzhou, China). The proteins were eluted with buffer containing 50 mM Tris-HCl and 10 mM reduced glutathione at pH 8.0, and peak fractions were collected, concentrated, and buffer-exchanged into PBS.

### 2.3. Epitope Screening by Western Blotting and ELISA

Purified GST-tagged truncated proteins were separated by 12% SDS-PAGE and transferred onto PVDF membranes. Western blotting with mAb N179 was performed as described above. The same protein samples were subjected to a separate Western blot analysis following the same procedure, using an anti-GST monoclonal antibody (1:2000, Beyotime, Shanghai, China) to confirm the presence of GST-tagged proteins.

For ELISA, purified GST-tagged truncated proteins were quantified by BCA assay, diluted to 1 μg/mL in carbonate–bicarbonate coating buffer (pH 9.6), and added to 96-well plates at 100 μL per well. After overnight coating at 4 °C, the plates were blocked with 5% BSA in TBST and incubated with mAb N179 (1:10,000), followed by HRP-conjugated goat anti-mouse IgG (1:2000). TMB substrate was then added, and the reaction was stopped before measuring OD_450_ (Beyotime, Shanghai, China).

GST protein expressed from the empty vector was used as the negative-protein control, with three replicate wells included in each independent ELISA experiment. Wells containing coating buffer alone served as uncoated blank controls. For each independent experiment, the positivity cutoff was operationally defined as 2.1 times the mean OD_450_ value of the three GST negative-protein control wells. A similar 2.1-fold negative-control criterion has been used as an empirical operational threshold for distinguishing positive from negative reactivity in previous indirect ELISA studies, including monoclonal-antibody-based epitope characterization and recombinant-antigen evaluation [15,17]. Accordingly, this criterion was adopted here for qualitative classification of the reactivity of the truncation fragments. The blank controls were used to monitor nonspecific background signal and were not included in the calculation of the positivity cutoff. A truncated fragment was classified as positive when the mean OD_450_ value of its three technical replicate wells exceeded the experiment-specific cutoff. This threshold was used solely for epitope-mapping purposes and was not intended to represent a clinically validated diagnostic cutoff.

### 2.4. Sequence Retrieval and Multiple Sequence Alignment

To assess epitope conservation across representative SARS-CoV-2 lineages, 11 N protein sequences representing the reference strain and selected major SARS-CoV-2 lineages were retrieved from the NCBI GenBank database. In addition, the six-amino-acid QTVTLL motif was used as the query sequence for an NCBI BLAST (https://blast.ncbi.nlm.nih.gov/Blast.cgi, accessed on 25 August 2026) search against SARS-CoV-2 sequences to identify potential naturally occurring variations within this region. Only complete N protein sequences without ambiguous amino acid residues were included. The database was accessed on 25 August 2026.

To evaluate the sequence specificity of the QTVTLL epitope among related viruses, N or NP amino acid sequences from seven human coronaviruses, four influenza viruses, and five bat coronaviruses were retrieved from the NCBI databases. Human and bat coronaviruses were selected because of their phylogenetic relationship to SARS-CoV-2 and their relevance to coronavirus evolution, whereas influenza viruses were included as representative clinically important respiratory pathogens relevant to differential diagnosis. This panel was intended as a focused sequence-level comparison rather than a comprehensive survey of all respiratory viruses.

Multiple sequence alignments were performed using KEGG ClustalW (https://www.genome.jp/tools-bin/clustalw, accessed on 8 April 2026) with default parameters and visualized with ESPript 3.2. In addition, stringent local pairwise alignment of the ^390^QTVTLL^395^ motif against viral nucleoproteins was performed using EMBOSS WATER (https://www.ebi.ac.uk/jdispatcher/psa/emboss_water, accessed on 8 April 2026) with the PAM30 substitution matrix, a gap-opening penalty of 10, and a gap-extension penalty of 0.5.

### 2.5. Structural Predictions

The secondary structure of the ^390^QTVTLL^395^ region was predicted using PSIPRED (https://bioinf.cs.ucl.ac.uk/psipred, accessed on 8 April 2026), and its hydrophobicity was evaluated with ProtScale using the Kyte–Doolittle hydropathy scale (https://web.expasy.org/protscale/, accessed on 8 April 2026) with a window size of 9. Linear B-cell epitope propensity was assessed using BepiPred 3.0 (https://services.healthtech.dtu.dk/services/BepiPred-3.0/, accessed on 8 April 2026). Surface exposure of the ^390^QTVTLL^395^ region was analyzed using the AlphaFold-predicted structural model of the full-length N protein (AlphaFold Database accession no. P0DTC9 (AF-0000000365840321-v1), corresponding to Universal Protein Resource (UniProt) P0DTC9, https://alphafold.ebi.ac.uk/entry/AF-0000000365840321, accessed on 8 April 2026). The confidence of the predicted local structure was evaluated using the per-residue predicted local distance difference test (pLDDT) scores stored in the B-factor field of the downloaded AlphaFold PDB file. The pLDDT scores of residues Q390-L395 were extracted for analysis. The secondary-structure, hydropathy, and domain-annotation panels were generated from the outputs of PSIPRED, ProtScale, and ScanProsite (https://prosite.expasy.org/scanprosite/, accessed on 8 April 2026) using a custom Python 3.14.0 script. The per-residue BepiPred 3.0 scores for the C-terminal region were replotted using GraphPad Prism 8, whereas the AlphaFold-predicted structural model was visualized using PyMOL version 3.2.0a.

### 2.6. Data Analysis

Three independent ELISA experiments were performed on separate experimental occasions. Within each independent experiment, every truncated fragment was analyzed in three technical replicate wells. The mean OD_450_ value of the three technical replicates was used to determine the reactivity of each fragment within that experiment. Positivity was assessed independently in each experiment using the corresponding experiment-specific cutoff described above. The positive and negative classifications of all fragments were consistent across the three independent experiments. The results from one representative independent experiment are presented in the Results section, whereas the other two independent experiments are provided in Supplementary Figure S1 to demonstrate reproducibility. GraphPad Prism 8 (GraphPad Software, San Diego, CA, USA) was used for data summarization and graph preparation. No inferential statistical comparisons were performed.

## 3. Results

### 3.1. Validation of mAb N179 Recognition of Recombinant N Protein

**Figure 2:**
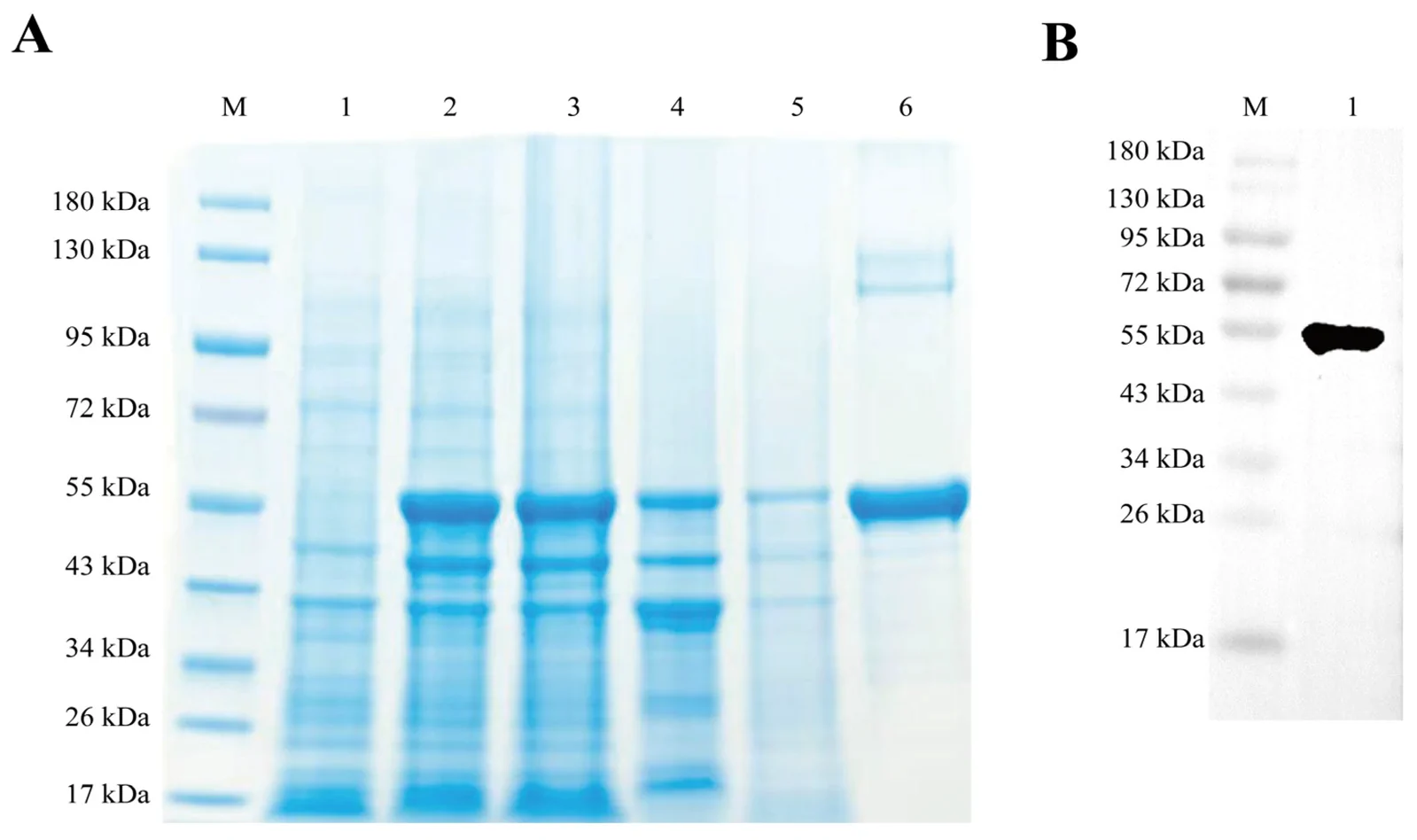
Expression, purification, and Western blot validation of recombinant SARS-CoV-2 N protein. (**A**) SDS-PAGE analysis of recombinant N protein expression and purification. Lane M, protein molecular weight marker; lane 1, whole-cell lysate before induction; lane 2, whole-cell lysate after IPTG induction; lane 3, supernatant fraction after ultrasonication; lane 4, pellet fraction after ultrasonication; lane 5, flow-through from the HisTrap Excel affinity column; lane 6, purified recombinant N protein after elution. (**B**) Western blot analysis of purified recombinant N protein probed with mAb N179. Lane M, protein molecular weight marker; lane 1, purified recombinant N protein.

To verify the binding specificity of mAb N179 for recombinant N protein, we expressed and purified His-tagged recombinant N protein (CorN) and analyzed it by Western blotting. SDS-PAGE analysis showed a distinct protein band at approximately 50 kDa following IPTG induction, corresponding to the expected molecular weight of CorN. This band was detected mainly in the supernatant fraction, indicating that CorN was expressed as a predominantly soluble protein (Figure 2A). Western blotting further confirmed that mAb N179 specifically recognized the purified CorN, yielding a single immunoreactive band at the expected molecular weight (Figure 2B).

### 3.2. Truncation Mapping Identifies Residues 390–395 as the Smallest Reactive Region Recognized by mAb N179

**Figure 3:**
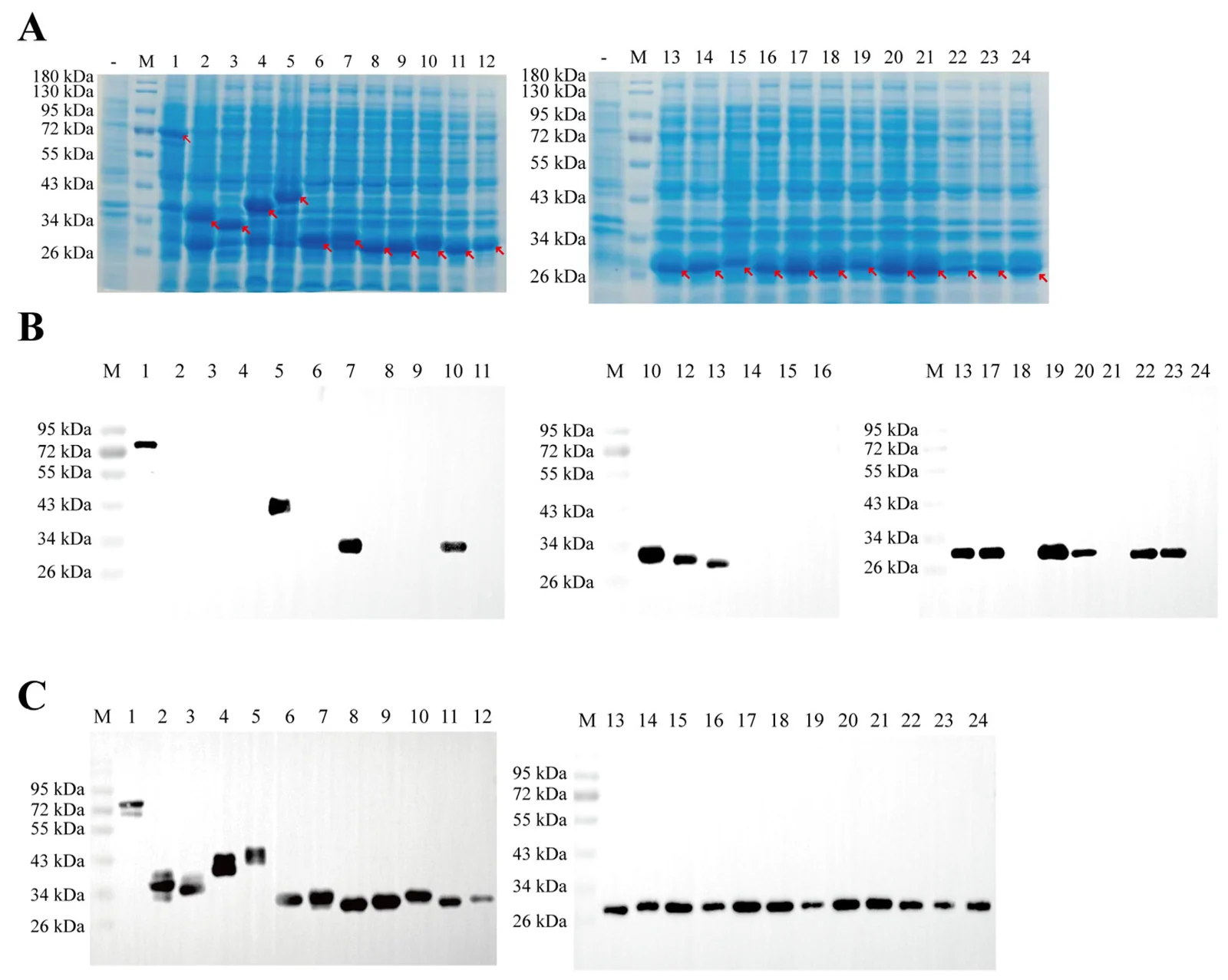
Western blot mapping of the antigenic epitope recognized by mAb N179. (**A**) Coomassie blue-stained SDS-PAGE gel showing the expression of each GST-tagged truncated protein fragment. Lane “-”, whole-cell lysate before induction; (**B**) Western blot analysis of the reactivity of the truncated fragments with mAb N179. Lane M, protein molecular weight marker; lane 1, 3–419 aa; lane 2, 3–98 aa; lane 3, 99–194 aa; lane 4, 177–304 aa; lane 5, 287–419 aa; lane 6, 355–387 aa; lane 7, 388–419 aa; lane 8, 355–369 aa; lane 9, 370–387 aa; lane 10, 388–405 aa; lane 11, 406–419 aa; lane 12, 388–401 aa; lane 13, 388–396 aa; lane 14, 397–405 aa; lane 15, 392–405 aa; lane 16, 392–401 aa; lane 17, 388–395 aa; lane 18, 388–394 aa; lane 19, 389–396 aa; lane 20, 389–395 aa; lane 21, 389–394 aa; lane 22, 390–396 aa; lane 23, 390–395 aa; lane 24, 391–395 aa. The smallest fragment retaining detectable reactivity with mAb N179 under the truncation-mapping conditions was 390–395 aa. (**C**) Western blot analysis using an anti-GST antibody, confirming the presence of GST-tagged proteins in all lanes.

To localize the epitope recognized by mAb N179, we expressed a series of GST-fused N protein fragments, including a near-full-length construct and progressively truncated C-terminal fragments. SDS-PAGE analysis showed that each truncated protein migrated as a distinct band at the expected molecular weight following IPTG induction, indicating successful expression of all constructs (Figure 3A).

Western blotting showed that mAb N179 recognized the near-full-length N protein fragment (3–419 aa) and the C-terminal fragments 287–419 aa and 388–419 aa, but did not react with the N-terminal fragment (3–98 aa) or the central-region fragments 99–194 aa and 177–304 aa. These results initially localized the epitope to the C-terminal region (388–419 aa) (Figure 3B, lanes 1–11). Further truncation showed that the 388–396 aa fragment retained reactivity with mAb N179, whereas the 397–405 aa, 392–405 aa, and 392–401 aa fragments showed no detectable immunoreactivity, further narrowing the reactive region to residues 388–396 (Figure 3B, lanes 12–16). Sequential single-residue truncation demonstrated that deletion of leucine at position 395 (L395; lane 21) or glutamine at position 390 (Q390; lane 24) abolished immunoreactivity. Thus, under the present truncation-mapping conditions, ^390^QTVTLL^395^ was the smallest fragment that retained detectable reactivity with mAb N179 (lane 23). Western blotting with an anti-GST monoclonal antibody further confirmed that all truncated proteins carried the GST tag (Figure 3C, lanes 1–24).

**Figure 4:**
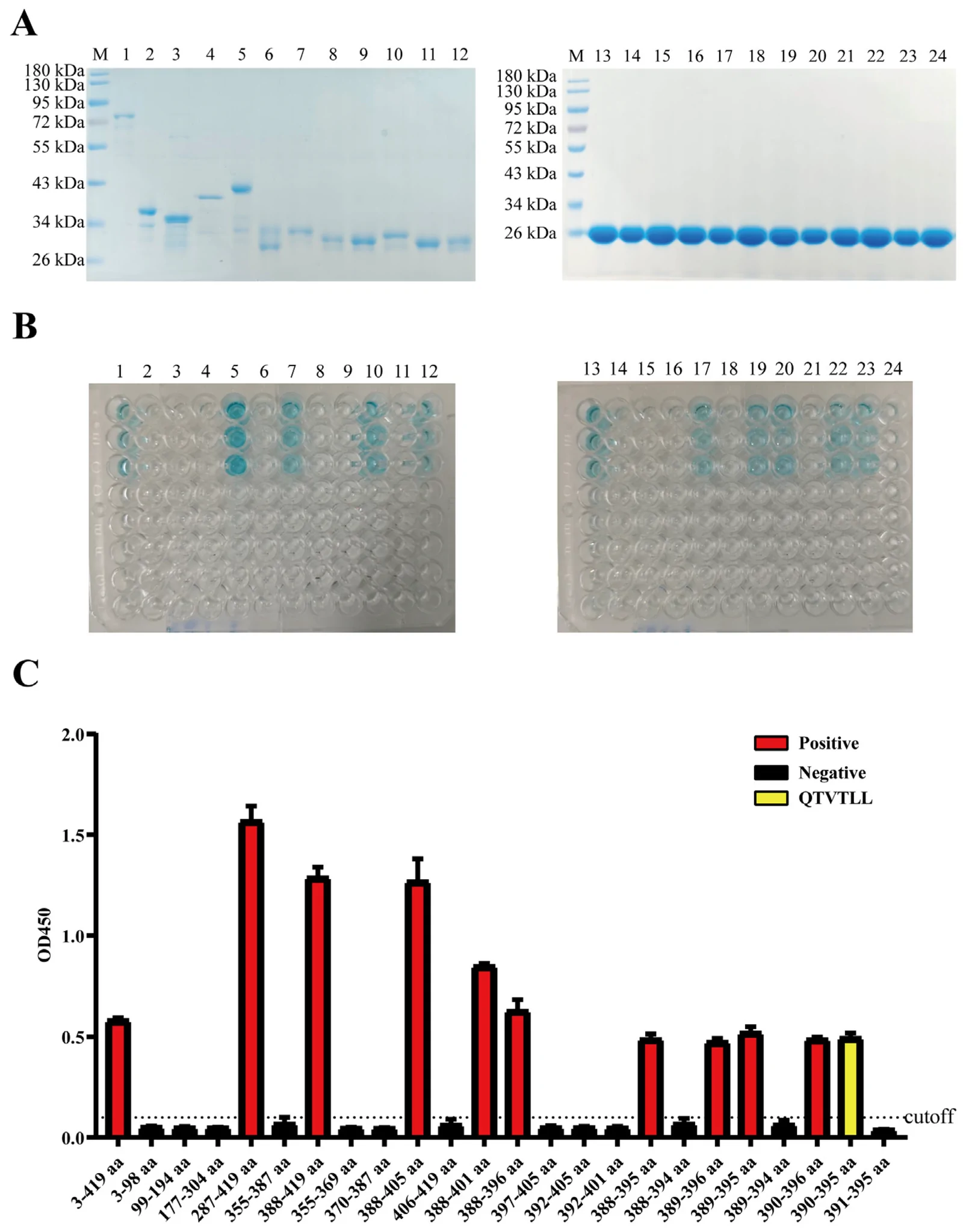
ELISA validation of the antigenic epitope recognized by mAb N179. (**A**) SDS-PAGE analysis of the purified GST-fused truncated protein fragments. (**B**) Representative ELISA color development with TMB substrate from one of three independent experiments. (**C**) OD_450_ values for each truncated fragment from the same representative experiment. “Lane M, protein molecular weight marker; lane 1–24 correspond to the fragments defined in Figure 3B. Data are presented as the mean ± SD of three technical replicate wells. GST protein expressed from the empty vector served as the negative-protein control, and coating-buffer-only wells served as uncoated blank controls. The dashed line indicates the experiment-specific positivity cutoff, calculated as 2.1 times the mean OD_450_ value of the three GST negative-protein control wells. The other two independent ELISA experiments are presented in Supplementary Figure S1, and the positive/negative classification of each fragment was consistent across all three independent experiments.

To quantitatively validate the epitope-mapping results, the same set of GST-tagged truncated proteins was purified by GST affinity chromatography (Figure 4A), quantified by BCA assay, and coated onto 96-well plates at 1 μg/mL for ELISA analysis (Figure 4B). Consistent with the Western blotting data, the 388–419 aa, 388–405 aa, and 388–396 aa fragments reacted with mAb N179 and produced mean OD_450_ values above the experiment-specific positivity cutoff in all three independent experiments. The same positive or negative classification pattern was reproduced in the other two independent ELISA experiments (Figure S1), supporting the reproducibility of the truncation-mapping results. In contrast, fragments lacking the intact QTVTLL motif, including the 397–405 aa, 389–394 aa, and 391–395 aa fragments, were classified as negative in all three independent experiments because their mean OD_450_ values remained below the experiment-specific cutoffs. In the fine-mapping analysis, the 390–395 aa fragment containing the intact QTVTLL motif was consistently classified as positive, whereas the 389–394 aa and 391–395 aa fragments produced signals comparable to those of the negative-protein controls. These ELISA results were consistent with the Western blotting findings and further supported the identification of QTVTLL (390–395 aa) as the smallest reactive region detected in the present truncation-mapping system (Figure 4C).

### 3.3. The QTVTLL Region Is Conserved Among the Representative SARS-CoV-2 Lineages Analyzed

**Figure 5:**
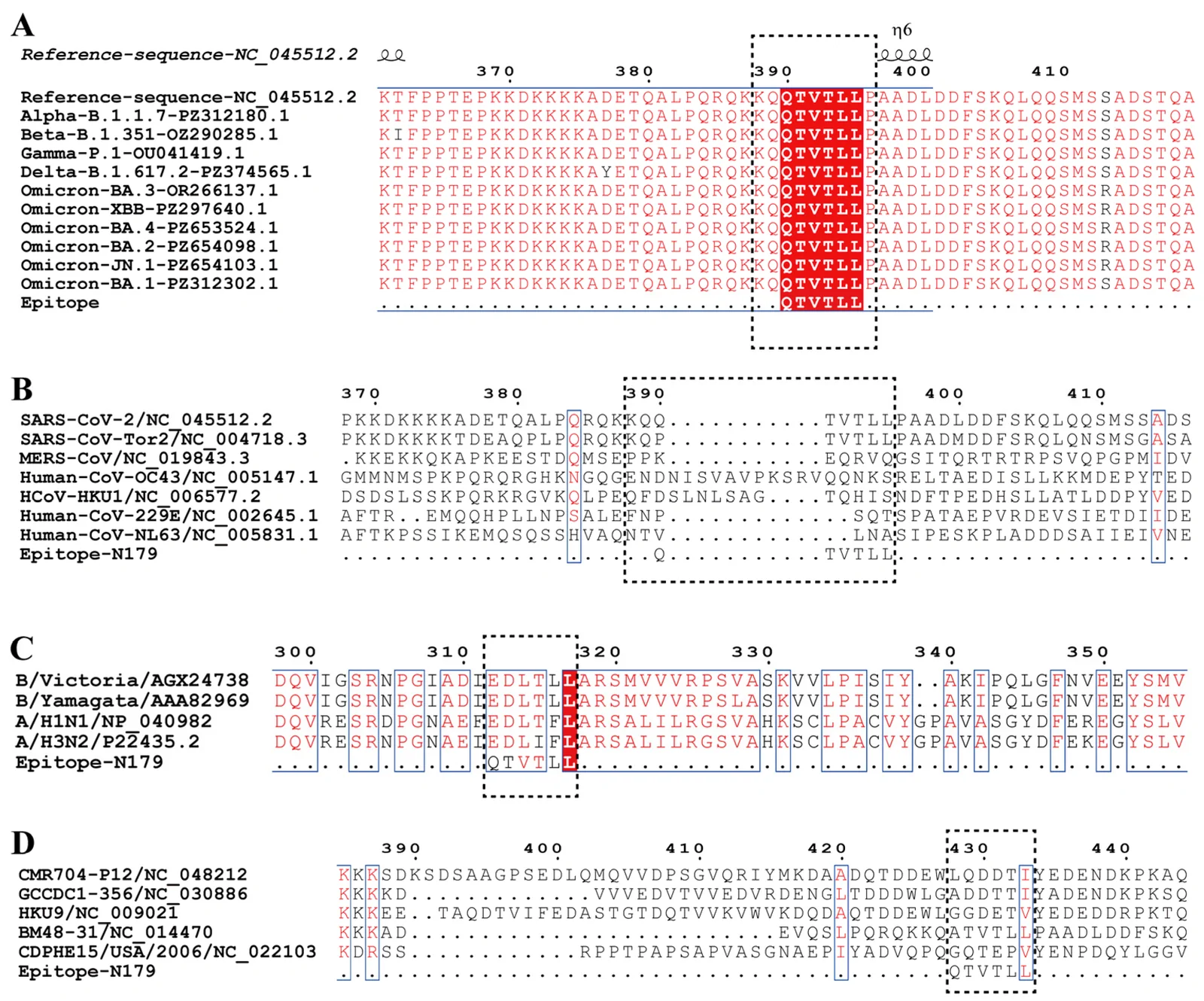
Conservation and sequence specificity of the ^390^QTVTLL^395^ epitope. (**A**) Multiple sequence alignment of 11 representative SARS-CoV-2 N protein sequences, including the reference sequence and the Alpha, Beta, Gamma, Delta, Omicron BA.1, BA.2, BA.3, BA.4, JN.1, and XBB lineages. The ^390^QTVTLL^395^ region is indicated by a dashed box and was completely conserved in all sequences analyzed. The secondary-structure annotation above the alignment was derived from the AlphaFold-predicted N protein model and indicates that the epitope lacks regular alpha-helical or beta-strand structure. (**B**–**D**) Comparative sequence analysis of the QTVTLL motif among selected respiratory viruses and bat coronaviruses. The dashed box indicates the region identical to QTVTLL in the alignment. (**B**) seven human coronaviruses, (**C**) four influenza viruses, and (**D**) five bat coronaviruses. Among the viral sequences included in this analysis, an exact QTVTLL sequence match was identified only in SARS-CoV-2. SARS-CoV Tor2 and bat coronavirus BM48-31 each contained a single substitution, corresponding to 5/6 identity, whereas no identical continuous six-residue motif was identified in the other viruses analyzed. Multiple sequence alignments were performed using KEGG ClustalW and visualized with ESPript 3.2.

Multiple sequence alignment showed that ^390^QTVTLL^395^ was completely conserved in the 11 SARS-CoV-2 sequences analyzed, including Alpha, Beta, Gamma, Delta, Omicron BA.1-BA.4, JN.1, and XBB (Figure 5A). These sequences represent major SARS-CoV-2 evolutionary lineages with different genetic backgrounds. Consistent with the representative lineage analysis, the NCBI BLAST search identified no naturally occurring amino acid substitutions within the QTVTLL motif among the SARS-CoV-2 sequences retrieved. The complete BLAST results are provided in Supplementary Figure S2. In contrast, no exact QTVTLL match was identified in the 15 selected non-SARS-CoV-2 nucleoprotein sequences examined (Figure 5B–D). SARS-CoV Tor2 and bat coronavirus BM48-31 contained PTVTLL and ATVTLL, respectively, each differing from the SARS-CoV-2 QTVTLL sequence by a single residue and therefore showing 5/6 identity. The remaining viruses showed only two or three identical residues in their best local alignments (33.3–50.0% identity; Table 1). Thus, the motif was conserved across the SARS-CoV-2 lineages analyzed and was sequence-distinct from the other human respiratory and bat viruses included in this study. In our previous study [16], the N179-based detection system was evaluated against several respiratory viruses, including influenza viruses and other human coronaviruses, and no positive cross-reactive signals were observed. These previous results, together with the sequence comparison performed in the current study, support the high specificity of N179 recognition and are consistent with the sequence-based analysis.

### 3.4. The QTVTLL Region Is Predicted to Lie Within a Flexible C-Terminal Region of the N Protein

**Figure 6:**
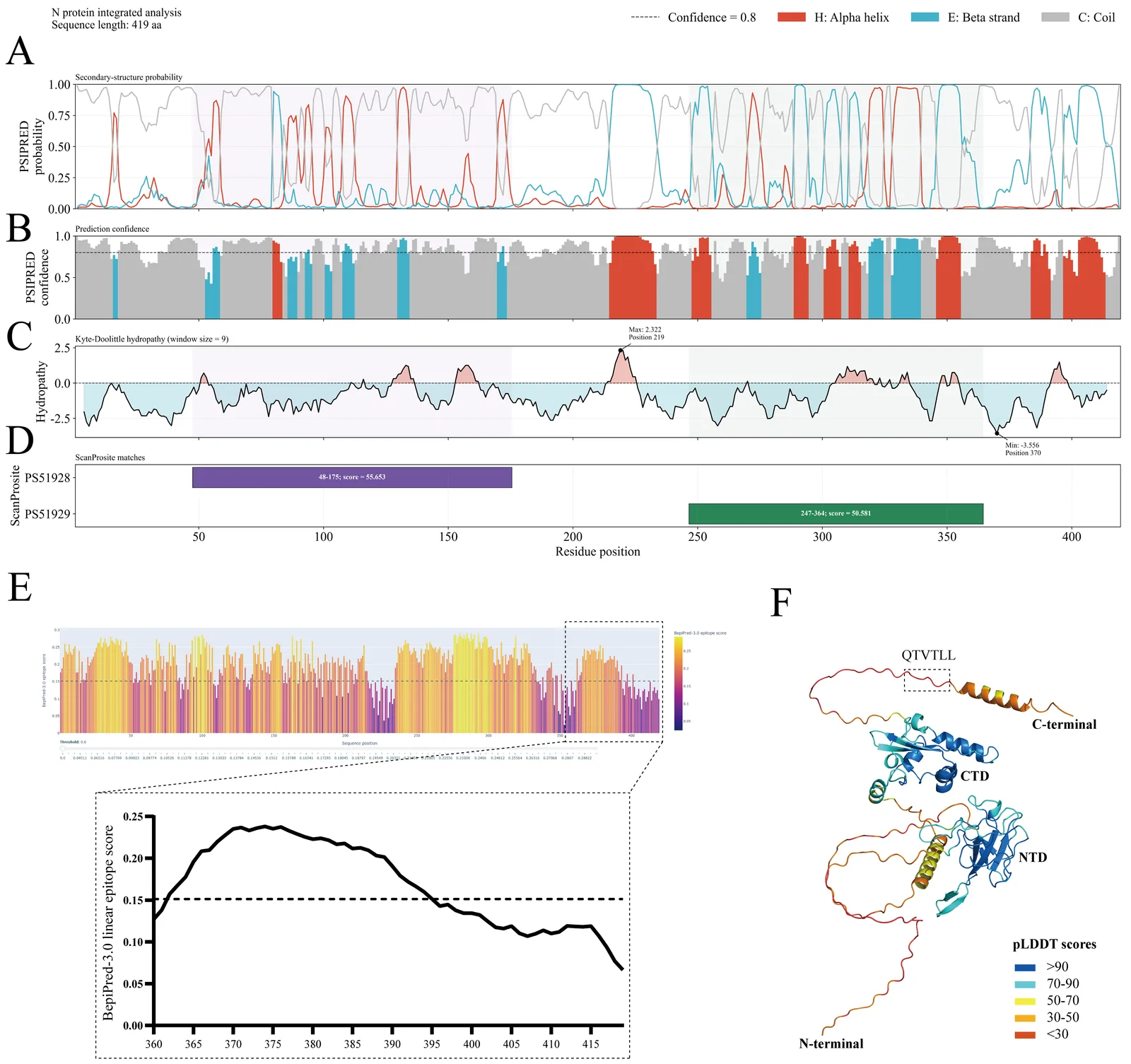
Predicted structural features of the ^390^QTVTLL^395^ epitope region. (**A**) Secondary structure probability distribution predicted by PSIPRED. (**B**) Secondary structure state assignment predicted by PSIPRED. (**C**) Kyte-Doolittle hydropathy plot generated with a window size of 9. (**D**) ScanProsite domain annotation showing the approximate positions of N-terminal domain (NTD) and C-terminal dimerization domain (CTD) of SARS-CoV-2 N protein. (**E**) Per-residue linear B-cell epitope scores predicted by BepiPred 3.0 for Q390-L395. (**F**) AlphaFold-predicted structural model of the full-length N protein visualized using PyMOL and colored according to the AlphaFold pLDDT confidence scale. The QTVTLL region is marked with a dashed box.

Structural prediction analyses indicated that the QTVTLL epitope is located in a flexible C-terminal region of the N protein that is predicted to lack regular secondary structure and lies downstream of the C-terminal dimerization domain (Figure 6). Secondary structure analysis showed that this segment was predicted to adopt a predominantly random-coil conformation (Figure 6A,B), and Kyte-Doolittle hydropathy analysis indicated a hydrophilic profile, consistent with potential surface exposure (Figure 6C). ScanProsite domain annotation placed the QTVTLL region outside the annotated N-terminal RNA-binding domain and C-terminal dimerization domain (CTD) (Figure 6D). Together with the secondary-structure prediction, this location is consistent with a flexible C-terminal tail. BepiPred 3.0 assigned per-residue scores of 0.1908, 0.1791, 0.1791, 0.1662, 0.1598, and 0.1515 to Q390, T391, V392, T393, L394, and L395, respectively. All six residues scored above the default threshold of 0.1512, although the score for L395 was only marginally above the cutoff (Figure 6E). The AlphaFold pLDDT scores for Q390, T391, V392, T393, L394, and L395 were 24.59, 25.30, 28.30, 27.86, 28.69, and 27.33, respectively, with a mean value of 27.01. All six residues therefore had pLDDT scores below 50, indicating very low confidence in the predicted local conformation of this C-terminal region. Accordingly, the AlphaFold model was used only to provide general structural context for the location of residues 390–395 within the C-terminal region (Figure 6F), and the specific local conformation and surface accessibility of the QTVTLL region cannot be reliably inferred from the AlphaFold model alone. Thus, the structural findings presented here should be regarded as predictive and do not experimentally demonstrate epitope accessibility or the conformation adopted during N179 binding.

## 4. Discussion

This study identifies ^390^QTVTLL^395^ as the smallest reactive region recognized by mAb N179 under the truncation mapping conditions used. This conclusion is supported by sequential truncation analysis combined with Western blotting and ELISA, which together narrowed N179 binding to a compact six-residue motif. These findings narrow the N179-reactive sequence from a broad C-terminal region to a six-residue segment, thereby providing sequence-level information that may help interpret the previously observed performance of the N179-based colloidal gold immunochromatographic test strip.

The diagnostic relevance of this finding lies in the fact that the performance of antibody-based antigen tests depends on the stability of the actual antibody-binding site, rather than on the overall conservation of the target antigen alone [18]. Although the N protein is generally more conserved than the spike protein, specific N-protein substitutions, including D399N and R203M, have been reported to impair the sensitivity of antigen detection assays [11,19]. These observations underscore the need to evaluate diagnostic antibodies at the epitope level. To address this issue, we evaluated three features relevant to N179 recognition: conservation of the N179-binding motif across representative SARS-CoV-2 lineages, sequence distinction from selected related respiratory viruses, and predicted structural features of the antibody-reactive region.

The predicted structural features of the ^390^QTVTLL^395^ region are consistent with, but do not experimentally demonstrate, potential accessibility to antibody recognition. Residues 390 to 395 are located downstream of the structured C-terminal dimerization domain, within the flexible C-terminal tail of the N protein [20]. Secondary-structure prediction, hydropathy analysis, and B-cell epitope propensity evaluation were consistent with a flexible region potentially compatible with antibody recognition. The AlphaFold model provided only general structural context because of the very low pLDDT scores for residues 390–395 and was therefore not used to infer local surface accessibility. This structural context is relevant because diagnostic antibodies targeting linear epitopes require access to their binding sites under antigen-detection conditions. The predicted flexibility of the C-terminal tail may be compatible with recognition of this short linear region by mAb N179, as observed for the recombinant N-protein fragments in Western blotting and ELISA. However, these structural interpretations should be considered predictive rather than definitive, because the structure of the mAb N179–epitope complex was not experimentally determined.

The identification of ^390^QTVTLL^395^ further refines the antigenic map of the SARS-CoV-2 N-protein C-terminal region. Several linear B-cell epitopes have previously been mapped to this region, including ^401^DFSKQLQQ^408^ and ^393^TLLPAADLDDFSKQL^407^. These reported epitopes range from 8 to 15 residues and partially overlap with, or lie adjacent to, the motif identified in the present study [21,22]. In comparison, ^390^QTVTLL^395^ represents the smallest six-residue linear region retaining detectable reactivity with mAb N179 under the present truncation-mapping conditions. The major implication of this finding is not simply that the epitope is shorter, but that mAb N179 recognition can now be assigned to a precisely delimited motif. This level of resolution allows variant-associated substitutions to be evaluated directly at the actual antibody-binding site rather than across a broader C-terminal antigenic region [15]. The loss of detectable immunoreactivity after truncation of Q390 or L395 indicates that the intact six-residue sequence is required for detectable binding within the present GST-fused truncation system. However, these experiments do not establish the energetic contribution or absolute requirement of each individual residue, which will require residue-level mutational analysis.

The conservation analysis provides important support for the diagnostic relevance of this motif. Multiple sequence alignment of 11 representative SARS-CoV-2 N protein sequences, including early variants such as Alpha and Beta as well as Omicron sub-lineages BA.1, BA.2, and BA.3, showed that ^390^QTVTLL^395^ was completely conserved across all variants analyzed. This finding is notable because several variants have accumulated substitutions elsewhere in the N protein, and previous reports have shown that certain N-protein mutations can affect antigen-test performance [15,19]. The absence of amino acid differences within residues 390–395 among the 11 representative sequences examined indicates that this region is conserved within the analyzed dataset. However, because the analysis was not exhaustive, these data do not establish absolute conservation across all SARS-CoV-2 sequences deposited in public databases. Whether this conservation reflects functional constraint or the limited number of sequences examined remains to be determined; nevertheless, it supports the possibility that N179 recognition is retained across the representative lineages included in this analysis.

The sequence comparison analysis further supports the conservation and distinctiveness of this motif among the viruses examined. Stringent local pairwise alignment using EMBOSS WATER with a PAM30 substitution matrix showed that a complete 6/6 match to ^390^QTVTLL^395^ was detected only in SARS-CoV-2. No identical continuous six-residue motif was identified in any of the six other human coronaviruses, four influenza viruses, or five bat coronaviruses examined [23]. SARS-CoV Tor2 and bat coronavirus BM48-31 each contained a single substitution at the first residue of the motif, resulting in a 5/6 match, whereas the remaining viruses showed only partial and discontinuous similarity in their best matching regions.

These data show that the ^390^QTVTLL^395^ motif is conserved among the SARS-CoV-2 lineages analyzed and differs from the best-matching regions of the selected non-SARS-CoV-2 viruses. However, sequence distinction should not be equated with experimentally demonstrated absence of antibody cross-reactivity. Although sequence analysis alone cannot exclude cross-reactivity against every possible respiratory virus or naturally occurring isolate, these findings provide a plausible sequence-level explanation for the previously observed lack of detectable cross-reactivity of the N179-based immunochromatographic assay with common respiratory pathogens.

This study has several limitations. The truncation-based mapping strategy identified the smallest reactive linear region recognized by N179 under the present experimental conditions, but it could not determine the contribution of individual residues or assess conformational determinants dependent on the native three-dimensional structure of the N protein [24]. Although computational predictions were consistent with potential accessibility of ^390^QTVTLL^395^, the N179-epitope complex was not structurally resolved [25,26], binding kinetics were not quantified, and residue-level contributions within the motif were not tested by alanine scanning or site-directed mutagenesis [27]. In particular, direct N179 binding to the 5/6-matched PTVTLL sequence of SARS-CoV Tor2 and ATVTLL sequence of bat coronavirus BM48-31 was not experimentally evaluated; therefore, potential cross-reactivity with these closely related motifs cannot be excluded. In addition, the sequence analysis was limited to representative SARS-CoV-2 lineages and selected related viruses. Future studies incorporating structural analysis, quantitative binding assays, residue-level mutagenesis, peptide competition or random peptide phage-display screening, experimental testing of closely related viral motifs, and broader variant surveillance would further clarify the sequence requirements, mutation tolerance, and diagnostic robustness of N179 [15].

In summary, this study identifies ^390^QTVTLL^395^ as the smallest reactive region recognized by mAb N179 under the truncation-mapping conditions used. This sequence was completely conserved among the 11 representative SARS-CoV-2 N protein sequences analyzed, and no identical six-residue sequence was identified among the selected non-SARS-CoV-2 viruses examined. Computational analyses further predicted that the region lies within a flexible C-terminal segment, although its local conformation and surface accessibility remain to be experimentally determined. These findings provide sequence-level information for understanding N179 recognition and support the QTVTLL region as a candidate reference sequence for further evaluation of diagnostic antibodies targeting the SARS-CoV-2 N protein. Broader sequence surveillance, quantitative binding measurements, residue-level mutational analysis, and experimental cross-reactivity testing will be required to determine the mutation tolerance and diagnostic robustness of N179.

## Figures and Tables

**Table 1:** Pairwise local alignment analysis of the ^390^QTVTLL^395^ motif across viral nucleoproteins using EMBOSS WATER.

Virus Strain	Complete Motif Match	* Identical Residues	Best-Matching Region
SARS-CoV-2	Yes	100% (6/6)	QTVTLL
SARS-CoV Tor2	No	83.3% (5/6)	PTVTLL
BM48-31	No	83.3% (5/6)	ATVTLL
GCCDC1	No	50.0% (3/6)	QKVTKQ
B/Victoria	No	50.0% (3/6)	EDLTLL
B/Yamagata	No	50.0% (3/6)	EDLTLL
HCoV-HKU1	No	50.0% (3/6)	QQVT--
HCoV-229E	No	50.0% (3/6)	-TVVL-
HCoV-OC43	No	50.0% (3/6)	-QTAT-
HCoV-NL63	No	50.0% (3/6)	--VTL-
HKU9-1	No	50.0% (3/6)	QTV---
MERS-CoV	No	33.3% (2/6)	-TV---
A/H1N1	No	33.3% (2/6)	QT----
A/H3N2	No	33.3% (2/6)	QT----
CDPHE15	No	33.3% (2/6)	QT----
CMR704-P12	No	33.3% (2/6)	QT----

Alignment parameters were as follows: PAM30 substitution matrix, gap opening penalty of 10, and gap extension penalty of 0.5. “Identical residues” indicates the number and percentage of residues identical to the six-residue QTVTLL query motif. “Best-matching region” indicates the amino acid segment that produced the highest alignment score. An exact 6/6 match was observed only in SARS-CoV-2; SARS-CoV, the closest relative, retained a single mismatched residue. * Identity was calculated as the number of residues identical to the six-residue query motif divided by six. Gap positions were counted as non-identical.

## Data Availability

The data presented in this study are available on request from the corresponding author.

## Supplementary Materials

The following supporting information can be downloaded at: https://www.mdpi.com/article/10.3390/v18090949/s1, Table S1. PCR primers used to amplify the SARS-CoV-2 N protein gene and truncated fragments; Figure S1. Independent ELISA validation of the reactivity of GST-fused truncated N-protein fragments with mAb N179. (A) Results of the second independent ELISA experiment. (B) Results of the third independent ELISA experiment. Each truncated fragment was analyzed in three technical replicate wells, and data are presented as the mean ± SD. GST protein expressed from the empty vector was used as the negative-protein control, with three replicate wells in each independent experiment, whereas coating-buffer-only wells served as uncoated blank controls. The dashed line in each panel represents the experiment-specific positivity cutoff, calculated as 2.1 times the mean OD450 value of the three GST negative-protein control wells from the corresponding experiment. Fragment classifications were consistent with those observed in the representative experiment shown in Figure 4; Figure S2. NCBI BLAST search of the SARS-CoV-2 QTVTLL motif. (A) NCBI BLAST search results using the six-amino-acid QTVTLL motif as the query sequence against SARS-CoV-2 sequences. (B) Detailed alignment information of representative BLAST hits. The retrieved SARS-CoV-2 sequences all contained the QTVTLL motif, and no naturally occurring amino acid substitutions were identified within this region.
