# Supplementary material for: Identification of a Conserved Linear Epitope in SARS-CoV-2 Nucleocapsid Protein Recognized by Monoclonal Antibody N179
Source: Viruses. 2026 Aug 30;18(9):949. doi: 10.3390/v18090949 (PMC13611893; doi:10.3390/v18090949)
Supplement: Supplementary file 1 [file viruses-18-00949-s001.zip › viruses-4502422-Supplementary File.pdf]

Table S1. PCR primers used to amplify the SARS-CoV-2 N protein gene and truncated fragments.

Fragment name	Forward primer (5′-3′)	Reverse primer (5′-3′)
3-419 aa	GATCTGGTTCCGCGTGGATCCGATAA TGGACCCCAAAATCAGCGA	TCAGTCAGTCACGATGCGGCCGCTTAGT GCTGACTCAACTCAGGCC
3-98 aa	GATCTGGTTCCGCGTGGATCCGATAA TGGACCCCAAAATCAGCGA	TCAGTCAGTCACGATGCGGCCGCTTAGT CACCACCACGAATTCGTCT
99-194 aa	GATCTGGTTCCGCGTGGATCCGGTAA AATGAAAGATCTCAGTCCAAGA	TCAGTCAGTCACGATGCGGCCGCTTATG AACTGTTGCGACTACGTGA
177-304 aa	GATCTGGTTCCGCGTGGATCCAGAGG CGGCAGTCAAGCC	TCAGTCAGTCACGATGCGGCCGCTTAAA TTTGCGGCCAATGTTTGTAATCA
287-419 aa	GATCTGGTTCCGCGTGGATCCGGGGA CCAGGAACTAATCAGAC	TCAGTCAGTCACGATGCGGCCGCTTAGT GCTGACTCAACTCAGGCC
355-387 aa	GATCTGGTTCCGCGTGGATCCAAGCA TATTGACGCATACAAAACATTCC	TCAGTCAGTCACGATGCGGCCGCTTATT TAGGCTCTGTTGGTGGGAATG
388-419 aa	GATCTGGTTCCGCGTGGATCCAAACA GCAAACTGTGACTCTTCTTCC	TCAGTCAGTCACGATGCGGCCGCTTAGT GCTGACTCAACTCAGGCC
355-369 aa	GATCTGGTTCCGCGTGGATCCAAGCA TATTGACGCATACAAAACATTCC	TCAGTCAGTCACGATGCGGCCGCTTATT TAGGCTCTGTTGGTGGGAAT
370-387 aa	GATCTGGTTCCGCGTGGATCCAAGGA CAAAAAGAAGAAGGCTGATGAA	TCAGTCAGTCACGATGCGGCCGCTTACT TCTGTCTCTGCGGTAAGGC
388-405 aa	GATCTGGTTCCGCGTGGATCCAAACA GCAAACTGTGACTCTTCTTCC	TCAGTCAGTCACGATGCGGCCGCTTATT TGGAGAAATCATCCAAATCTGCAGCA
406-419 aa	GATCTGGTTCCGCGTGGATCCCAATT GCAACAATCCATGAGCAGT	TCAGTCAGTCACGATGCGGCCGCTTAGT GCTGACTCAACTCAGGCC
388-396 aa	GATCTGGTTCCGCGTGGATCCAAACA GCAAACTGTGACTCTTCTT	TCAGTCAGTCACGATGCGGCCGCTTAAG GAAGAAGAGTCACAGTTTGCT
397-405 aa	GATCTGGTTCCGCGTGGATCCGCTGC AGATTTGGATGATTTCTCC	TCAGTCAGTCACGATGCGGCCGCTTATT TGGAGAAATCATCCAAATCTGCAGCA
388-401 aa	GATCTGGTTCCGCGTGGATCCAAACA GCAAACTGTGACTCTTCTTC	TCAGTCAGTCACGATGCGGCCGCTTAAT CCAAATCTGCAGCAGGAAGAAG
392-401 aa	GATCTGGTTCCGCGTGGATCCGTGAC TCTTCTTCCTGCTGCA	TCAGTCAGTCACGATGCGGCCGCTTAAT CCAAATCTGCAGCAGGAAGAAG
392-405 aa	GATCTGGTTCCGCGTGGATCCGTGAC TCTTCTTCCTGCTGCA	TCAGTCAGTCACGATGCGGCCGCTTATT TGGAGAAATCATCCAAATCTGCAGCA
*N-388-395 aa	GATCCAAACAGCAAACTGTGACTCTT CTTTAAGC	GGCCGCTTAAAGAAGAGTCACAGTTTG CTGTTTG
*N-388-394 aa	GATCCAAACAGCAAACTGTGACTCTT TAAGC	GGCCGCTTAAAGAGTCACAGTTTGCTGT TTG
*N-389-396 aa	GATCCCAGCAAACTGTGACTCTTCTT CCTTAAGC	GGCCGCTTAAGGAAGAAGAGTCACAGT TTGCTGG
*N-389-395 aa	GATCCCAGCAAACTGTGACTCTTCTT TAAGC	GGCCGCTTAAAGAAGAGTCACAGTTTG CTGG
*N-389-394 aa	GATCCCAGCAAACTGTGACTCTTTAA	GGCCGCTTAAAGAGTCACAGTTTGCTGG

	GC	
*N-390-396 aa	GATCCCAAACTGTGACTCTTCTTCCTT AAGC	GGCCGCTTAAGGAAGAAGAGTCACAGT TTGG
*N-390-395 aa	GATCCCAAACTGTGACTCTTCTTTAA GC	GGCCGCTTAAAGAAGAGTCACAGTTTG G
*N-391-395 aa	GATCCACTGTGACTCTTCTTTAAGC	GGCCGCTTAAAGAAGAGTCACAGTG

* Fragments with the "N-" prefix were generated by direct primers annealing.

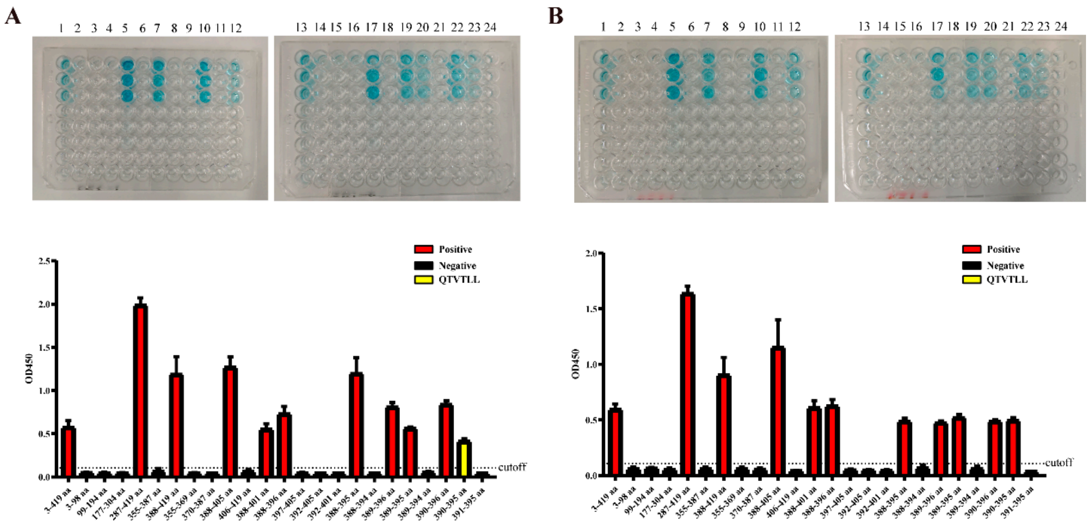

Figure S1. Independent ELISA validation of the reactivity of GST-fused truncated N-protein fragments with mAb N179. (A) Results of the second independent ELISA experiment. (B) Results of the third independent ELISA experiment. Each truncated fragment was analyzed in three technical replicate wells, and data are presented as the mean ± SD. GST protein expressed from the empty vector was used as the negative-protein control, with three replicate wells in each independent experiment, whereas coating-buffer-only wells served as uncoated blank controls. The dashed line in each panel represents the experiment-specific positivity cutoff, calculated as 2.1 times the mean OD_{450} value of the three GST negative-protein control wells from the corresponding experiment. Fragment classifications were consistent with those observed in the representative experiment shown in Figure 4.

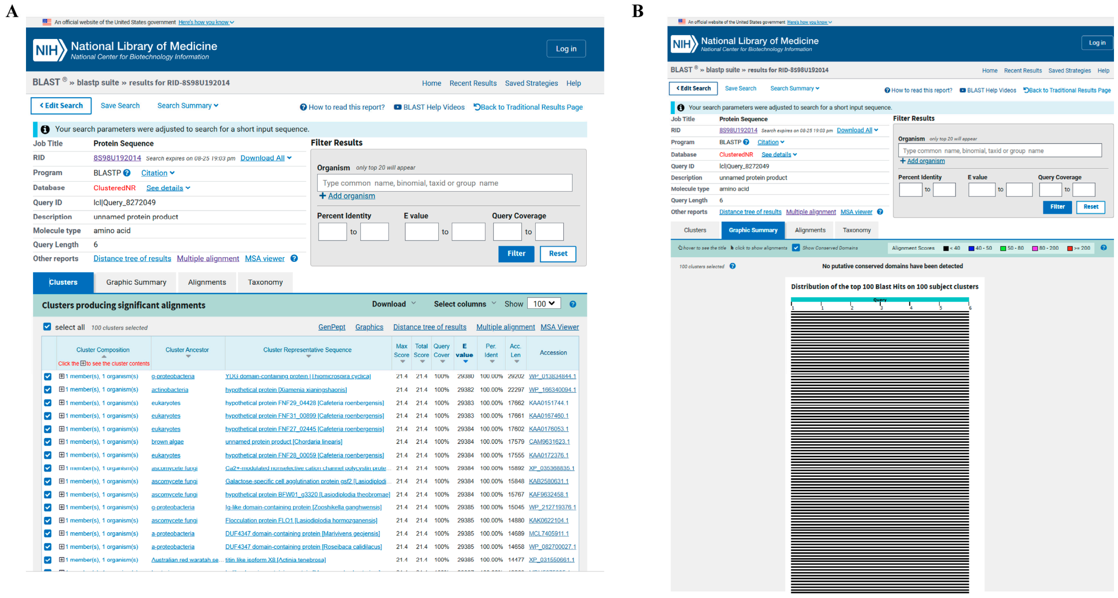

Figure S2. NCBI BLAST search of the SARS-CoV-2 QTVTLL motif. (A) NCBI BLAST search results using the six-amino-acid QTVTLL motif as the query sequence against SARS-CoV-2 sequences. (B) Detailed alignment information of representative BLAST hits. The retrieved SARS-CoV-2 sequences all contained the QTVTLL motif, and no naturally occurring amino acid substitutions were identified within this region.
